# Sudden-Onset Obsessive-Compulsive Disorder in a Pediatric Patient: A Case-Based Literature Review on PANDAS and Immunomodulatory Treatment Strategies

**DOI:** 10.1192/j.eurpsy.2026.11234

**Published:** 2026-07-31

**Authors:** C. Lopez Montealegre, G. Cabeza, C. Cañete, E. Acosta, I. Salmeron, S. A. Gutierrez De Larraya

**Affiliations:** Hospital Clinic de Barcelona, Barcelona, Spain

## Abstract

**Introduction:**

Pediatric Autoimmune Neuropsychiatric Disorders Associated with Streptococcal infections (PANDAS) describe a subset of prepubertal children who develop an abrupt onset of obsessive-compulsive symptoms and/or tics following a Group A Streptococcus (GAS) infection. Although the diagnosis remains controversial, the proposed pathophysiological mechanism involves an autoimmune response in genetically susceptible individuals, where molecular mimicry leads to the production of antibodies that cross-react with antigens in the basal ganglia. Nevertheless, this hypothesis has not been conclusively confirmed in current literature. Treatment is typically symptomatic, however, there is potential for clinical improvement with immunomodulatory and antibiotic therapies in selected cases.

**Objectives:**

To describe a pediatric case of sudden-onset OCD with suspected autoimmune etiology and favorable response to antibiotic treatment, and to review the current literature on therapeutic strategies for PANDAS, including antibiotics and immunomodulatory interventions.

**Methods:**

A narrative literature review was conducted using PubMed with the following terms: (“PANDAS” OR “PANS”) AND (“Obsessive-Compulsive Disorder” OR “OCD”) AND (“Antibiotic treatment” OR “Immunomodulatory therapy”). Twelve research studies involving treatments such as penicillin, azithromycin, intravenous immunoglobulin (IVIG), plasma exchange, tonsillectomy, cognitive behavioral therapy (CBT), NSAIDs, and corticosteroids met inclusion criteria. Additionally, 65 case reports describing the use of antibiotics, immunomodulators, and/or psychotropics were identified.

**Results:**

Twelve research studies met inclusion criteria, evaluating treatments such as penicillin, azithromycin, intravenous immunoglobulin (IVIG), plasma exchange, tonsillectomy, cognitive behavioral therapy (CBT), NSAIDs, and corticosteroids. Additionally, 65 case reports described the use of antibiotics, immunomodulatory therapies, and/or psychotropic medications. While antibiotics and IVIG showed potential benefit in selected cases, the overall quality of evidence was low, with high risk of bias and lack of randomized controlled trials. Corticosteroids and NSAIDs were used in acute phases with variable outcomes, and plasma exchange was reserved for severe, refractory presentations. CBT remained a consistent component of symptom management across studies.

**Conclusions:**

This case supports the hypothesis of an autoimmune mechanism in a subset of pediatric OCD presentations. Although various immunomodulatory strategies have been explored, including antibiotics, IVIG, corticosteroids, NSAIDs, and plasma exchange, rigorously conducted research remains scarce. Further investigation is needed to establish standardized diagnostic criteria and evidence-based treatment protocols for PANDAS and related autoimmune neuropsychiatric syndromes.

**Disclosure of Interest:**

None Declared

